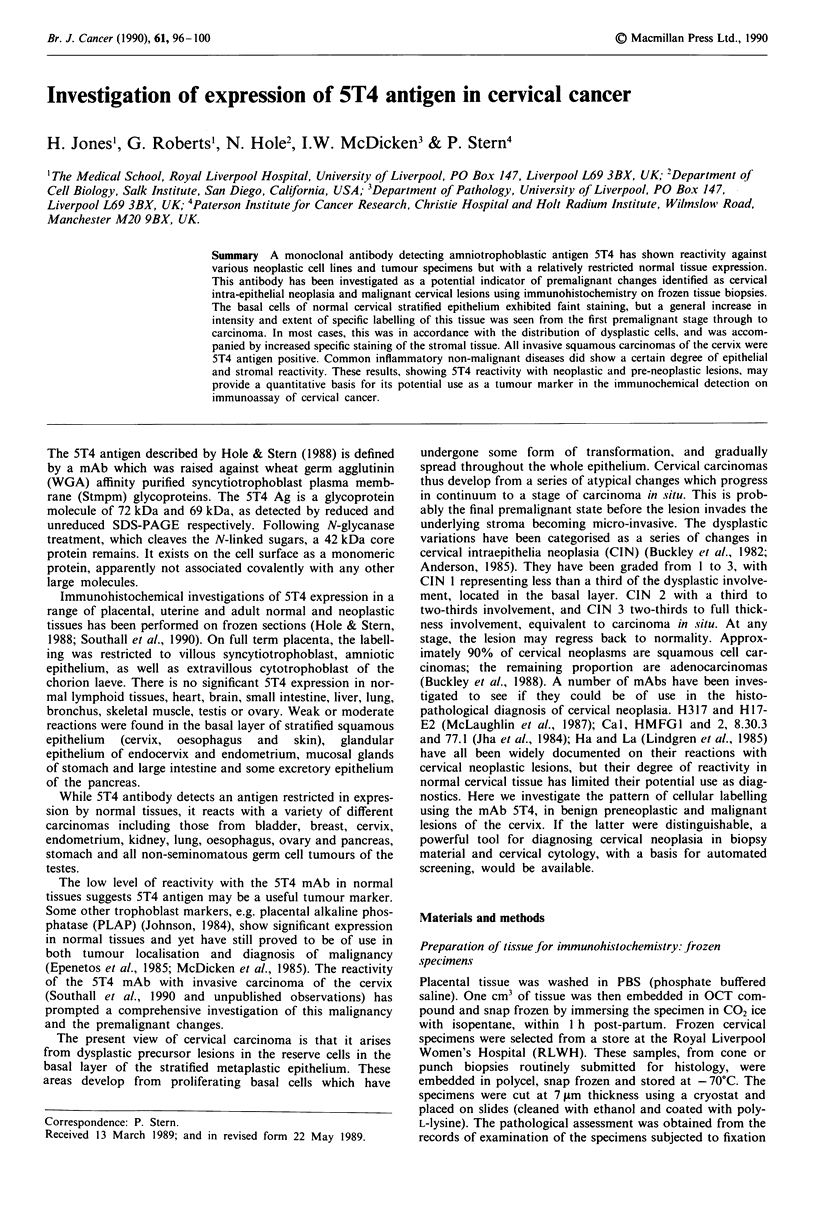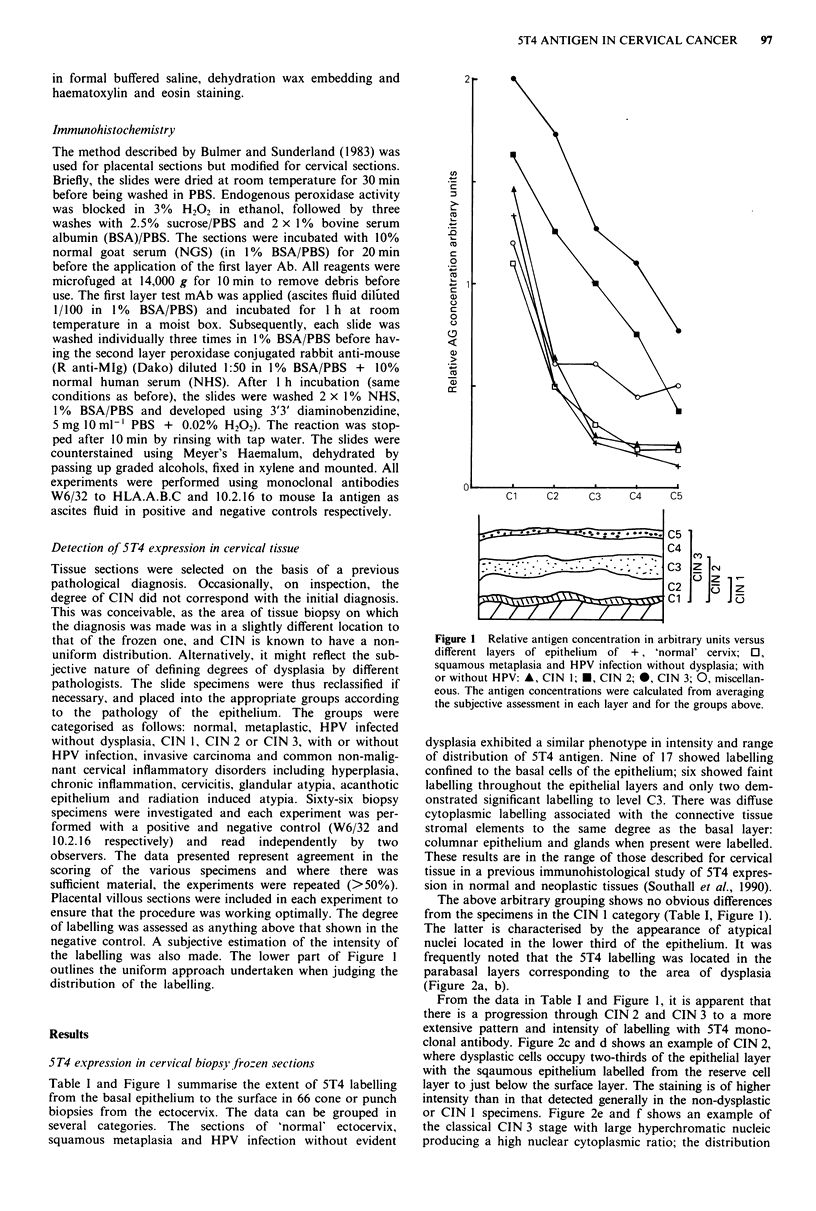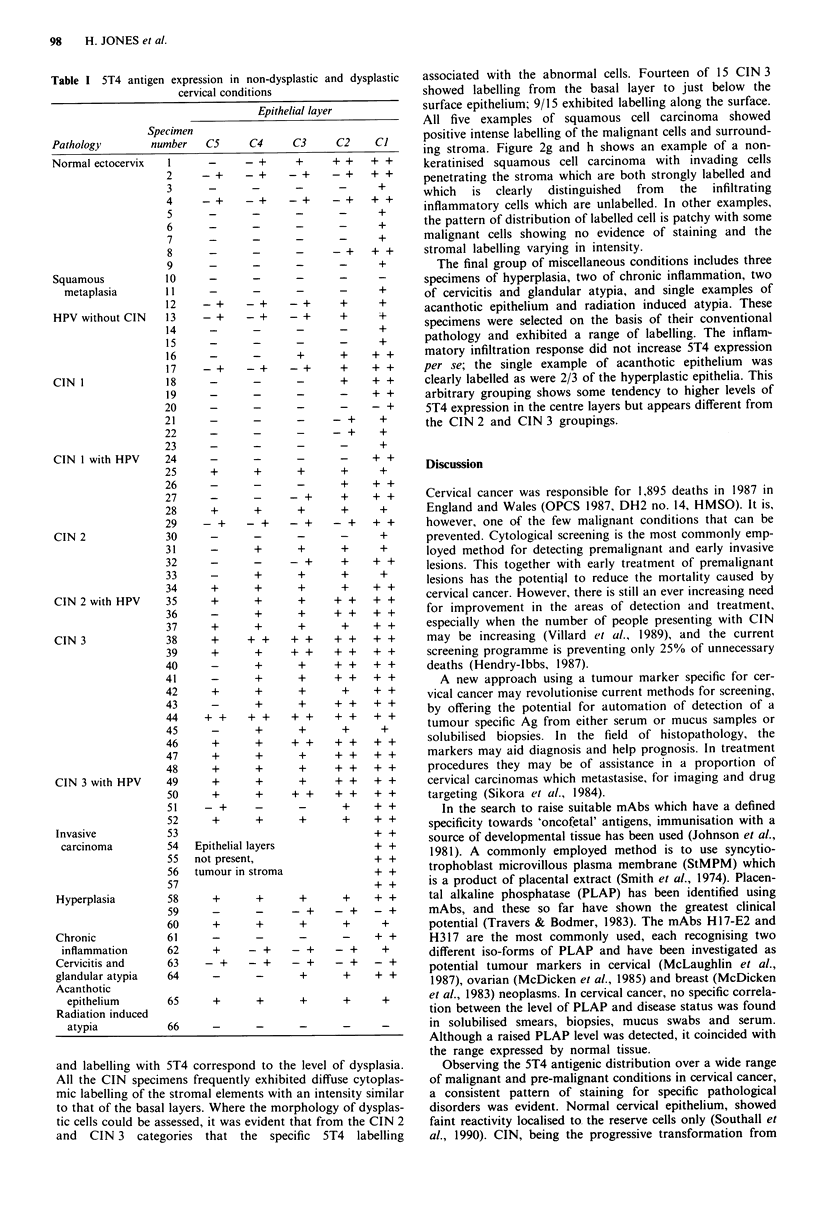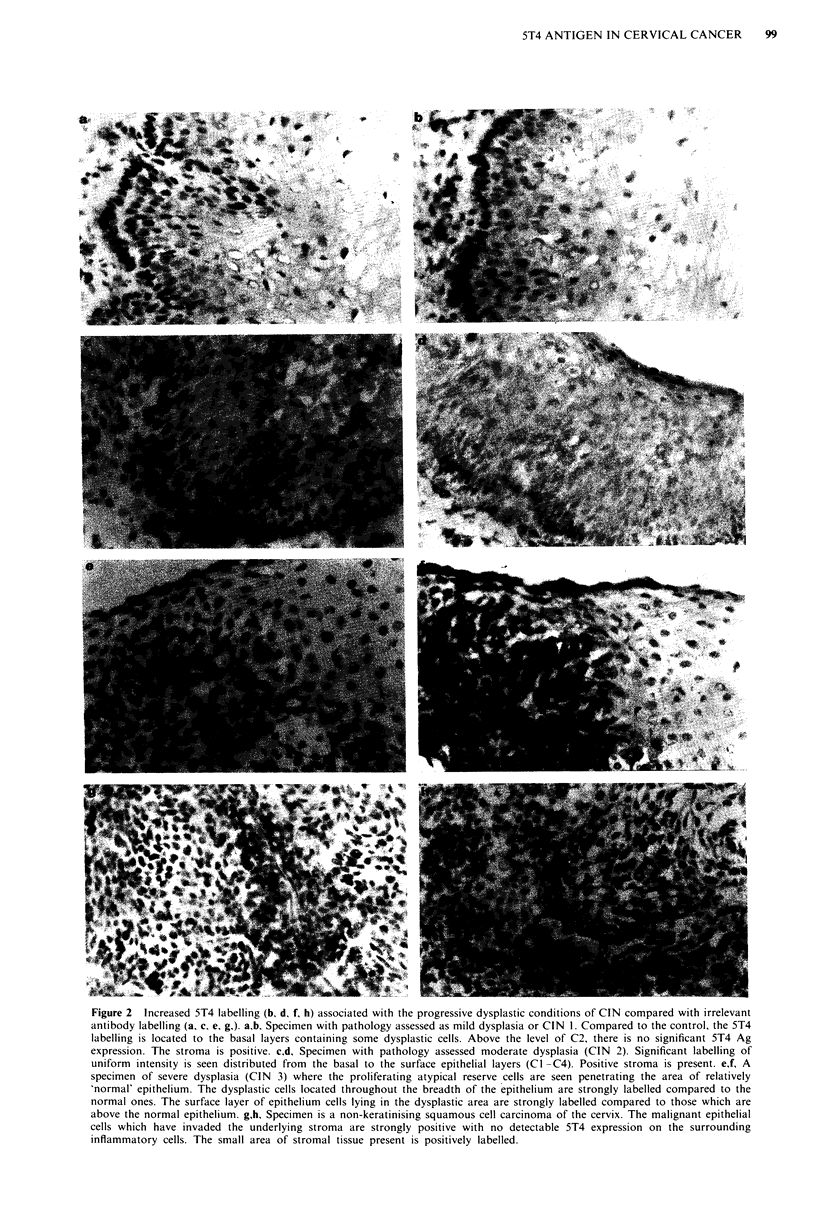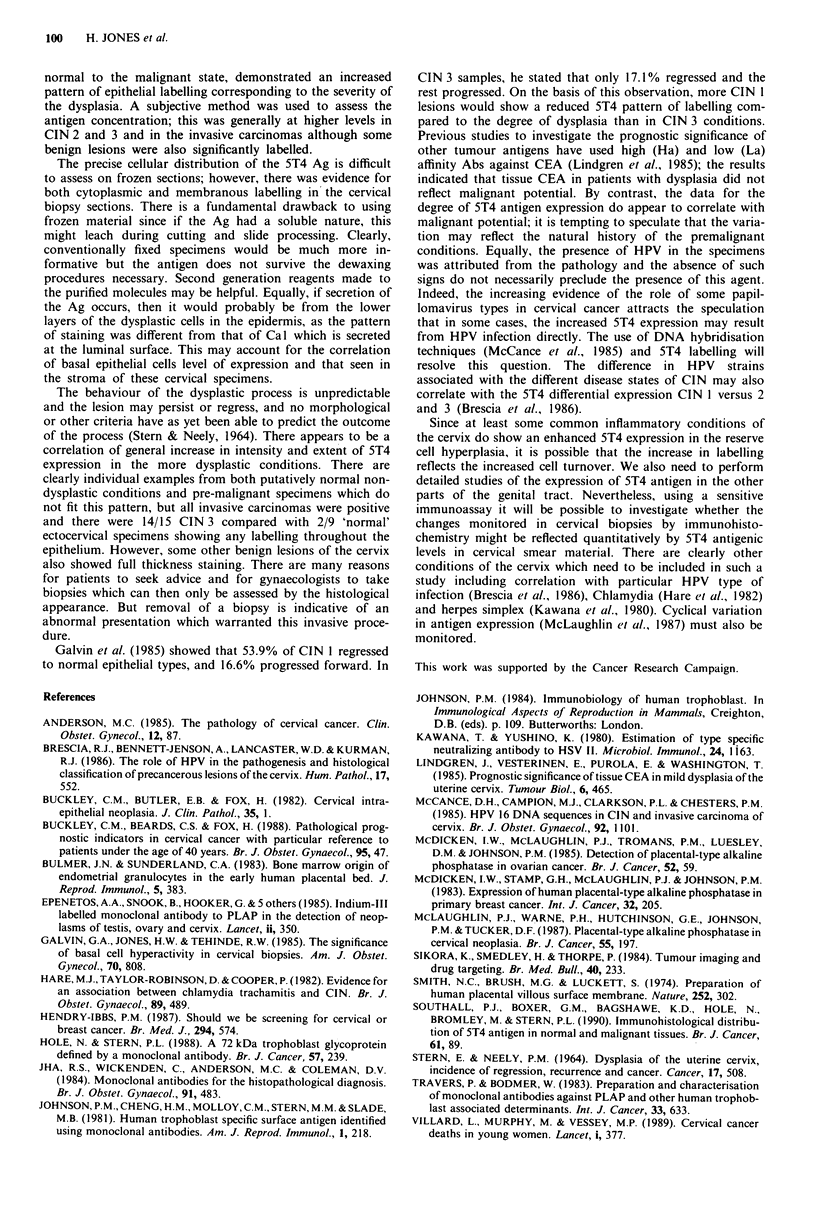# Investigation of expression of 5T4 antigen in cervical cancer.

**DOI:** 10.1038/bjc.1990.21

**Published:** 1990-01

**Authors:** H. Jones, G. Roberts, N. Hole, I. W. McDicken, P. Stern

**Affiliations:** Medical School, Royal Liverpool Hospital, University of Liverpool, UK.

## Abstract

**Images:**


*Br. J. Cancer* (1990), **61**, 96–100 © Macmillan Press Ltd., 1990

### Investigation of expression of 5T4 antigen in cervical cancer

H. Jones[1], G. Roberts[1], N. Hole[2], I.W. McDicken[3] & P. Stern[4]

[1]*The Medical School, Royal Liverpool Hospital, University of Liverpool, PO Box 147, Liverpool L69 3BX, UK;* [2]*Department of Cell Biology, Salk Institute, San Diego, California, USA;* [3]*Department of Pathology, University of Liverpool, PO Box 147, Liverpool L69 3BX, UK;* [4]*Paterson Institute for Cancer Research, Christie Hospital and Holt Radium Institute, Wilmslow Road, Manchester M20 9BX, UK.*

**Summary** A monoclonal antibody detecting amniotrophoblastic antigen 5T4 has shown reactivity against various neoplastic cell lines and tumour specimens but with a relatively restricted normal tissue expression. This antibody has been investigated as a potential indicator of premalignant changes identified as cervical intra-epithelial neoplasia and malignant cervical lesions using immunohistochemistry on frozen tissue biopsies. The basal cells of normal cervical stratified epithelium exhibited faint staining, but a general increase in intensity and extent of specific labelling of this tissue was seen from the first premalignant stage through to carcinoma. In most cases, this was in accordance with the distribution of dysplastic cells, and was accompanied by increased specific staining of the stromal tissue. All invasive squamous carcinomas of the cervix were 5T4 antigen positive. Common inflammatory non-malignant diseases did show a certain degree of epithelial and stromal reactivity. These results, showing 5T4 reactivity with neoplastic and pre-neoplastic lesions, may provide a quantitative basis for its potential use as a tumour marker in the immunochemical detection on immunoassay of cervical cancer.

The 5T4 antigen described by Hole & Stern (1988) is defined by a mAb which was raised against wheat germ agglutinin (WGA) affinity purified syncytiotrophoblast plasma membrane (Stmpm) glycoproteins. The 5T4 Ag is a glycoprotein molecule of 72 kDa and 69 kDa, as detected by reduced and unreduced SDS-PAGE respectively. Following *N*-glycanase treatment, which cleaves the *N*-linked sugars, a 42 kDa core protein remains. It exists on the cell surface as a monomeric protein, apparently not associated covalently with any other large molecules.

Immunohistochemical investigations of 5T4 expression in a range of placental, uterine and adult normal and neoplastic tissues has been performed on frozen sections (Hole & Stern, 1988; Southall *et al.*, 1990). On full term placenta, the labelling was restricted to villous syncytiotrophoblast, amniotic epithelium, as well as extravillous cytotrophoblast of the chorion laeve. There is no significant 5T4 expression in normal lymphoid tissues, heart, brain, small intestine, liver, lung, bronchus, skeletal muscle, testis or ovary. Weak or moderate reactions were found in the basal layer of stratified squamous epithelium (cervix, oesophagus and skin), glandular epithelium of endocervix and endometrium, mucosal glands of stomach and large intestine and some excretory epithelium of the pancreas.

While 5T4 antibody detects an antigen restricted in expression by normal tissues, it reacts with a variety of different carcinomas including those from bladder, breast, cervix, endometrium, kidney, lung, oesophagus, ovary and pancreas, stomach and all non-seminomatous germ cell tumours of the testes.

The low level of reactivity with the 5T4 mAb in normal tissues suggests 5T4 antigen may be a useful tumour marker. Some other trophoblast markers, e.g. placental alkaline phosphatase (PLAP) (Johnson, 1984), show significant expression in normal tissues and yet have still proved to be of use in both tumour localisation and diagnosis of malignancy (Epenetos *et al.*, 1985; McDicken *et al.*, 1985). The reactivity of the 5T4 mAb with invasive carcinoma of the cervix (Southall *et al.*, 1990 and unpublished observations) has prompted a comprehensive investigation of this malignancy and the premalignant changes.

The present view of cervical carcinoma is that it arises from dysplastic precursor lesions in the reserve cells in the basal layer of the stratified metaplastic epithelium. These areas develop from proliferating basal cells which have undergone some form of transformation, and gradually spread throughout the whole epithelium. Cervical carcinomas thus develop from a series of atypical changes which progress in continuum to a stage of carcinoma *in situ*. This is probably the final premalignant state before the lesion invades the underlying stroma becoming micro-invasive. The dysplastic variations have been categorised as a series of changes in cervical intraepithelia neoplasia (CIN) (Buckley *et al.*, 1982; Anderson, 1985). They have been graded from 1 to 3, with CIN 1 representing less than a third of the dysplastic involvement, located in the basal layer. CIN 2 with a third to two-thirds involvement, and CIN 3 two-thirds to full thickness involvement, equivalent to carcinoma *in situ*. At any stage, the lesion may regress back to normality. Approximately 90% of cervical neoplasms are squamous cell carcinomas; the remaining proportion are adenocarcinomas (Buckley *et al.*, 1988). A number of mAbs have been investigated to see if they could be of use in the histopathological diagnosis of cervical neoplasia. H317 and H17-E2 (McLaughlin *et al.*, 1987); Ca1, HMFG1 and 2, 8.30.3 and 77.1 (Jha *et al.*, 1984); Ha and La (Lindgren *et al.*, 1985) have all been widely documented on their reactions with cervical neoplastic lesions, but their degree of reactivity in normal cervical tissue has limited their potential use as diagnostics. Here we investigate the pattern of cellular labelling using the mAb 5T4, in benign preneoplastic and malignant lesions of the cervix. If the latter were distinguishable, a powerful tool for diagnosing cervical neoplasia in biopsy material and cervical cytology, with a basis for automated screening, would be available.

### Materials and methods

### *Preparation of tissue for immunohistochemistry: frozen specimens*

Placental tissue was washed in PBS (phosphate buffered saline). One $cm^3$ of tissue was then embedded in OCT compound and snap frozen by immersing the specimen in $CO_2$ ice with isopentane, within 1 h post-partum. Frozen cervical specimens were selected from a store at the Royal Liverpool Women's Hospital (RLWH). These samples, from cone or punch biopsies routinely submitted for histology, were embedded in polycel, snap frozen and stored at −70°C. The specimens were cut at 7 μm thickness using a cryostat and placed on slides (cleaned with ethanol and coated with poly-L-lysine). The pathological assessment was obtained from the records of examination of the specimens subjected to fixation

Correspondence: P. Stern.
Received 13 March 1989; and in revised form 22 May 1989.

in formal buffered saline, dehydration wax embedding and haematoxylin and eosin staining.

*Immunohistochemistry*

The method described by Bulmer and Sunderland (1983) was used for placental sections but modified for cervical sections. Briefly, the slides were dried at room temperature for 30 min before being washed in PBS. Endogenous peroxidase activity was blocked in 3% $H_2O_2$ in ethanol, followed by three washes with 2.5% sucrose/PBS and 2 x 1% bovine serum albumin (BSA)/PBS. The sections were incubated with 10% normal goat serum (NGS) (in 1% BSA/PBS) for 20 min before the application of the first layer Ab. All reagents were microfuged at 14,000 *g* for 10 min to remove debris before use. The first layer test mAb was applied (ascites fluid diluted 1/100 in 1% BSA/PBS) and incubated for 1 h at room temperature in a moist box. Subsequently, each slide was washed individually three times in 1% BSA/PBS before having the second layer peroxidase conjugated rabbit anti-mouse (R anti-MIg) (Dako) diluted 1:50 in 1% BSA/PBS + 10% normal human serum (NHS). After 1 h incubation (same conditions as before), the slides were washed 2 x 1% NHS, 1% BSA/PBS and developed using 3'3' diaminobenzidine, 5 mg 10 $ml^{-1}$ PBS + 0.02% $H_2O_2$). The reaction was stopped after 10 min by rinsing with tap water. The slides were counterstained using Meyer's Haemalum, dehydrated by passing up graded alcohols, fixed in xylene and mounted. All experiments were performed using monoclonal antibodies W6/32 to HLA.A.B.C and 10.2.16 to mouse Ia antigen as ascites fluid in positive and negative controls respectively.

*Detection of 5T4 expression in cervical tissue*

Tissue sections were selected on the basis of a previous pathological diagnosis. Occasionally, on inspection, the degree of CIN did not correspond with the initial diagnosis. This was conceivable, as the area of tissue biopsy on which the diagnosis was made was in a slightly different location to that of the frozen one, and CIN is known to have a non-uniform distribution. Alternatively, it might reflect the subjective nature of defining degrees of dysplasia by different pathologists. The slide specimens were thus reclassified if necessary, and placed into the appropriate groups according to the pathology of the epithelium. The groups were categorised as follows: normal, metaplastic, HPV infected without dysplasia, CIN 1, CIN 2 or CIN 3, with or without HPV infection, invasive carcinoma and common non-malignant cervical inflammatory disorders including hyperplasia, chronic inflammation, cervicitis, glandular atypia, acanthotic epithelium and radiation induced atypia. Sixty-six biopsy specimens were investigated and each experiment was performed with a positive and negative control (W6/32 and 10.2.16 respectively) and read independently by two observers. The data presented represent agreement in the scoring of the various specimens and where there was sufficient material, the experiments were repeated (>50%). Placental villous sections were included in each experiment to ensure that the procedure was working optimally. The degree of labelling was assessed as anything above that shown in the negative control. A subjective estimation of the intensity of the labelling was also made. The lower part of Figure 1 outlines the uniform approach undertaken when judging the distribution of the labelling.

### Results

*5T4 expression in cervical biopsy frozen sections*

Table I and Figure 1 summarise the extent of 5T4 labelling from the basal epithelium to the surface in 66 cone or punch biopsies from the ectocervix. The data can be grouped in several categories. The sections of 'normal' ectocervix, squamous metaplasia and HPV infection without evident

**Figure 1** Relative antigen concentration in arbitrary units versus different layers of epithelium of +, 'normal' cervix; □, squamous metaplasia and HPV infection without dysplasia; with or without HPV: ▲, CIN 1; ■, CIN 2; ●, CIN 3; O, miscellaneous. The antigen concentrations were calculated from averaging the subjective assessment in each layer and for the groups above.

dysplasia exhibited a similar phenotype in intensity and range of distribution of 5T4 antigen. Nine of 17 showed labelling confined to the basal cells of the epithelium; six showed faint labelling throughout the epithelial layers and only two demonstrated significant labelling to level C3. There was diffuse cytoplasmic labelling associated with the connective tissue stromal elements to the same degree as the basal layer: columnar epithelium and glands when present were labelled. These results are in the range of those described for cervical tissue in a previous immunohistological study of 5T4 expression in normal and neoplastic tissues (Southall *et al.*, 1990).

The above arbitrary grouping shows no obvious differences from the specimens in the CIN 1 category (Table I, Figure 1). The latter is characterised by the appearance of atypical nuclei located in the lower third of the epithelium. It was frequently noted that the 5T4 labelling was located in the parabasal layers corresponding to the area of dysplasia (Figure 2a, b).

From the data in Table I and Figure 1, it is apparent that there is a progression through CIN 2 and CIN 3 to a more extensive pattern and intensity of labelling with 5T4 monoclonal antibody. Figure 2c and d shows an example of CIN 2, where dysplastic cells occupy two-thirds of the epithelial layer with the sqaumous epithelium labelled from the reserve cell layer to just below the surface layer. The staining is of higher intensity than in that detected generally in the non-dysplastic or CIN 1 specimens. Figure 2e and f shows an example of the classical CIN 3 stage with large hyperchromatic nucleic producing a high nuclear cytoplasmic ratio; the distribution

**Table I** 5T4 antigen expression in non-dysplastic and dysplastic cervical conditions

| | | Epithelial layer | | | | |
|---|---|---|---|---|---|---|
| *Pathology* | *Specimen number* | *C5* | *C4* | *C3* | *C2* | *C1* |
| Normal ectocervix | 1 | – | – + | + | + + | + + |
| | 2 | – + | – + | – + | – + | + + |
| | 3 | – | – | – | – | + |
| | 4 | – + | – + | – + | – + | + + |
| | 5 | – | – | – | – | + |
| | 6 | – | – | – | – | + |
| | 7 | – | – | – | – | + |
| | 8 | – | – | – | – + | + + |
| | 9 | – | – | – | – | + |
| Squamous metaplasia | 10 | – | – | – | – | – |
| | 11 | – | – | – | – | + |
| | 12 | – + | – + | – + | + | + |
| HPV without CIN | 13 | – + | – + | – + | + | + |
| | 14 | – | – | – | – | + |
| | 15 | – | – | – | – | + |
| | 16 | – | – | + | + | + + |
| | 17 | – + | – + | – + | + | + + |
| CIN 1 | 18 | – | – | – | + | + + |
| | 19 | – | – | – | – | + + |
| | 20 | – | – | – | – | – + |
| | 21 | – | – | – | – + | + |
| | 22 | – | – | – | – + | + |
| | 23 | – | – | – | – | + |
| CIN 1 with HPV | 24 | – | – | – | – | + + |
| | 25 | + | + | + | + | + |
| | 26 | – | – | – | + | + + |
| | 27 | – | – | – + | + | + + |
| | 28 | + | + | + | + | + |
| | 29 | – + | – + | – + | – + | + + |
| CIN 2 | 30 | – | – | – | – | + |
| | 31 | – | + | + | + | + |
| | 32 | – | – | – + | + | + + |
| | 33 | – | + | + | + | + |
| | 34 | + | + | + | + | + + |
| CIN 2 with HPV | 35 | + | + | + | + + | + + |
| | 36 | – | + | + | + + | + + |
| | 37 | + | + | + | + | + + |
| CIN 3 | 38 | + | + + | + + | + + | + + |
| | 39 | + | + | + + | + + | + + |
| | 40 | – | + | + | + + | + + |
| | 41 | – | + | + | + + | + + |
| | 42 | + | + | + | + | + + |
| | 43 | – | + | + | + + | + + |
| | 44 | + + | + + | + + | + + | + + |
| | 45 | – | + | + | + | + |
| | 46 | + | + | + + | + + | + + |
| | 47 | + | + | + | + + | + + |
| | 48 | + | + | + | + + | + + |
| CIN 3 with HPV | 49 | + | + | + | + + | + + |
| | 50 | + | + | + + | + + | + + |
| | 51 | – + | – | – | + | + + |
| | 52 | + | + | + | + | + + |
| Invasive carcinoma | 53 | | | | | + + |
| | 54 | Epithelial layers | | | | + + |
| | 55 | not present, | | | | + + |
| | 56 | tumour in stroma | | | | + + |
| | 57 | | | | | + + |
| Hyperplasia | 58 | + | + | + | + | + + |
| | 59 | – | – | – + | – + | – + |
| | 60 | + | + | + | + | + |
| Chronic inflammation | 61 | – | – | – | – | + + |
| | 62 | + | – + | – + | – + | + |
| Cervicitis and glandular atypia | 63 | – + | – + | – + | – + | – + |
| | 64 | – | – | + | + | + + |
| Acanthotic epithelium | 65 | + | + | + | + | + |
| Radiation induced atypia | 66 | – | – | – | – | – |

and labelling with 5T4 correspond to the level of dysplasia. All the CIN specimens frequently exhibited diffuse cytoplasmic labelling of the stromal elements with an intensity similar to that of the basal layers. Where the morphology of dysplastic cells could be assessed, it was evident that from the CIN 2 and CIN 3 categories that the specific 5T4 labelling associated with the abnormal cells. Fourteen of 15 CIN 3 showed labelling from the basal layer to just below the surface epithelium; 9/15 exhibited labelling along the surface. All five examples of squamous cell carcinoma showed positive intense labelling of the malignant cells and surrounding stroma. Figure 2g and h shows an example of a non-keratinised squamous cell carcinoma with invading cells penetrating the stroma which are both strongly labelled and which is clearly distinguished from the infiltrating inflammatory cells which are unlabelled. In other examples, the pattern of distribution of labelled cell is patchy with some malignant cells showing no evidence of staining and the stromal labelling varying in intensity.

The final group of miscellaneous conditions includes three specimens of hyperplasia, two of chronic inflammation, two of cervicitis and glandular atypia, and single examples of acanthotic epithelium and radiation induced atypia. These specimens were selected on the basis of their conventional pathology and exhibited a range of labelling. The inflammatory infiltration response did not increase 5T4 expression *per se*; the single example of acanthotic epithelium was clearly labelled as were 2/3 of the hyperplastic epithelia. This arbitrary grouping shows some tendency to higher levels of 5T4 expression in the centre layers but appears different from the CIN 2 and CIN 3 groupings.

### Discussion

Cervical cancer was responsible for 1,895 deaths in 1987 in England and Wales (OPCS 1987, DH2 no. 14, HMSO). It is, however, one of the few malignant conditions that can be prevented. Cytological screening is the most commonly employed method for detecting premalignant and early invasive lesions. This together with early treatment of premalignant lesions has the potential to reduce the mortality caused by cervical cancer. However, there is still an ever increasing need for improvement in the areas of detection and treatment, especially when the number of people presenting with CIN may be increasing (Villard *et al.*, 1989), and the current screening programme is preventing only 25% of unnecessary deaths (Hendry-Ibbs, 1987).

A new approach using a tumour marker specific for cervical cancer may revolutionise current methods for screening, by offering the potential for automation of detection of a tumour specific Ag from either serum or mucus samples or solubilised biopsies. In the field of histopathology, the markers may aid diagnosis and help prognosis. In treatment procedures they may be of assistance in a proportion of cervical carcinomas which metastasise, for imaging and drug targeting (Sikora *et al.*, 1984).

In the search to raise suitable mAbs which have a defined specificity towards 'oncofetal' antigens, immunisation with a source of developmental tissue has been used (Johnson *et al.*, 1981). A commonly employed method is to use syncytiotrophoblast microvillous plasma membrane (StMPM) which is a product of placental extract (Smith *et al.*, 1974). Placental alkaline phosphatase (PLAP) has been identified using mAbs, and these so far have shown the greatest clinical potential (Travers & Bodmer, 1983). The mAbs H17-E2 and H317 are the most commonly used, each recognising two different iso-forms of PLAP and have been investigated as potential tumour markers in cervical (McLaughlin *et al.*, 1987), ovarian (McDicken *et al.*, 1985) and breast (McDicken *et al.*, 1983) neoplasms. In cervical cancer, no specific correlation between the level of PLAP and disease status was found in solubilised smears, biopsies, mucus swabs and serum. Although a raised PLAP level was detected, it coincided with the range expressed by normal tissue.

Observing the 5T4 antigenic distribution over a wide range of malignant and pre-malignant conditions in cervical cancer, a consistent pattern of staining for specific pathological disorders was evident. Normal cervical epithelium, showed faint reactivity localised to the reserve cells only (Southall *et al.*, 1990). CIN, being the progressive transformation from

**Figure 2** Increased 5T4 labelling (**b**, **d**, **f**, **h**) associated with the progressive dysplastic conditions of CIN compared with irrelevant antibody labelling (**a**, **c**, **e**, **g**,). **a**,**b**, Specimen with pathology assessed as mild dysplasia or CIN 1. Compared to the control, the 5T4 labelling is located to the basal layers containing some dysplastic cells. Above the level of C2, there is no significant 5T4 Ag expression. The stroma is positive. **c**,**d**, Specimen with pathology assessed moderate dysplasia (CIN 2). Significant labelling of uniform intensity is seen distributed from the basal to the surface epithelial layers (C1–C4). Positive stroma is present. **e**,**f**, A specimen of severe dysplasia (CIN 3) where the proliferating atypical reserve cells are seen penetrating the area of relatively 'normal' epithelium. The dysplastic cells located throughout the breadth of the epithelium are strongly labelled compared to the normal ones. The surface layer of epithelium cells lying in the dysplastic area are strongly labelled compared to those which are above the normal epithelium. **g**,**h**, Specimen is a non-keratinising squamous cell carcinoma of the cervix. The malignant epithelial cells which have invaded the underlying stroma are strongly positive with no detectable 5T4 expression on the surrounding inflammatory cells. The small area of stromal tissue present is positively labelled.

normal to the malignant state, demonstrated an increased pattern of epithelial labelling corresponding to the severity of the dysplasia. A subjective method was used to assess the antigen concentration; this was generally at higher levels in CIN 2 and 3 and in the invasive carcinomas although some benign lesions were also significantly labelled.

The precise cellular distribution of the 5T4 Ag is difficult to assess on frozen sections; however, there was evidence for both cytoplasmic and membranous labelling in the cervical biopsy sections. There is a fundamental drawback to using frozen material since if the Ag had a soluble nature, this might leach during cutting and slide processing. Clearly, conventionally fixed specimens would be much more informative but the antigen does not survive the dewaxing procedures necessary. Second generation reagents made to the purified molecules may be helpful. Equally, if secretion of the Ag occurs, then it would probably be from the lower layers of the dysplastic cells in the epidermis, as the pattern of staining was different from that of Ca1 which is secreted at the luminal surface. This may account for the correlation of basal epithelial cells level of expression and that seen in the stroma of these cervical specimens.

The behaviour of the dysplastic process is unpredictable and the lesion may persist or regress, and no morphological or other criteria have as yet been able to predict the outcome of the process (Stern & Neely, 1964). There appears to be a correlation of general increase in intensity and extent of 5T4 expression in the more dysplastic conditions. There are clearly individual examples from both putatively normal non-dysplastic conditions and pre-malignant specimens which do not fit this pattern, but all invasive carcinomas were positive and there were 14/15 CIN 3 compared with 2/9 'normal' ectocervical specimens showing any labelling throughout the epithelium. However, some other benign lesions of the cervix also showed full thickness staining. There are many reasons for patients to seek advice and for gynaecologists to take biopsies which can then only be assessed by the histological appearance. But removal of a biopsy is indicative of an abnormal presentation which warranted this invasive procedure.

Galvin et al. (1985) showed that 53.9% of CIN 1 regressed to normal epithelial types, and 16.6% progressed forward. In CIN 3 samples, he stated that only 17.1% regressed and the rest progressed. On the basis of this observation, more CIN 1 lesions would show a reduced 5T4 pattern of labelling compared to the degree of dysplasia than in CIN 3 conditions. Previous studies to investigate the prognostic significance of other tumour antigens have used high (Ha) and low (La) affinity Abs against CEA (Lindgren et al., 1985); the results indicated that tissue CEA in patients with dysplasia did not reflect malignant potential. By contrast, the data for the degree of 5T4 antigen expression do appear to correlate with malignant potential; it is tempting to speculate that the variation may reflect the natural history of the premalignant conditions. Equally, the presence of HPV in the specimens was attributed from the pathology and the absence of such signs do not necessarily preclude the presence of this agent. Indeed, the increasing evidence of the role of some papillomavirus types in cervical cancer attracts the speculation that in some cases, the increased 5T4 expression may result from HPV infection directly. The use of DNA hybridisation techniques (McCance et al., 1985) and 5T4 labelling will resolve this question. The difference in HPV strains associated with the different disease states of CIN may also correlate with the 5T4 differential expression CIN 1 versus 2 and 3 (Brescia et al., 1986).

Since at least some common inflammatory conditions of the cervix do show an enhanced 5T4 expression in the reserve cell hyperplasia, it is possible that the increase in labelling reflects the increased cell turnover. We also need to perform detailed studies of the expression of 5T4 antigen in the other parts of the genital tract. Nevertheless, using a sensitive immunoassay it will be possible to investigate whether the changes monitored in cervical biopsies by immunohistochemistry might be reflected quantitatively by 5T4 antigenic levels in cervical smear material. There are clearly other conditions of the cervix which need to be included in such a study including correlation with particular HPV type of infection (Brescia et al., 1986), Chlamydia (Hare et al., 1982) and herpes simplex (Kawana et al., 1980). Cyclical variation in antigen expression (McLaughlin et al., 1987) must also be monitored.

This work was supported by the Cancer Research Campaign.

### References

ANDERSON, M.C. (1985). The pathology of cervical cancer. *Clin. Obstet. Gynecol.*, **12**, 87.

BRESCIA, R.J., BENNETT-JENSON, A., LANCASTER, W.D. & KURMAN, R.J. (1986). The role of HPV in the pathogenesis and histological classification of precancerous lesions of the cervix. *Hum. Pathol.*, **17**, 552.

BUCKLEY, C.M., BUTLER, E.B. & FOX, H. (1982). Cervical intra-epithelial neoplasia. *J. Clin. Pathol.*, **35**, 1.

BUCKLEY, C.M., BEARDS, C.S. & FOX, H. (1988). Pathological prognostic indicators in cervical cancer with particular reference to patients under the age of 40 years. *Br. J. Obstet. Gynaecol.*, **95**, 47.

BULMER, J.N. & SUNDERLAND, C.A. (1983). Bone marrow origin of endometrial granulocytes in the early human placental bed. *J. Reprod. Immunol.*, **5**, 383.

EPENETOS, A.A., SNOOK, B., HOOKER, G. & 5 others (1985). Indium-III labelled monoclonal antibody to PLAP in the detection of neoplasms of testis, ovary and cervix. *Lancet*, **ii**, 350.

GALVIN, G.A., JONES, H.W. & TEHINDE, R.W. (1985). The significance of basal cell hyperactivity in cervical biopsies. *Am. J. Obstet. Gynecol.*, **70**, 808.

HARE, M.J., TAYLOR-ROBINSON, D. & COOPER, P. (1982). Evidence for an association between chlamydia trachamitis and CIN. *Br. J. Obstet. Gynaecol.*, **89**, 489.

HENDRY-IBBS, P.M. (1987). Should we be screening for cervical or breast cancer. *Br. Med. J.*, **294**, 574.

HOLE, N. & STERN, P.L. (1988). A 72 kDa trophoblast glycoprotein defined by a monoclonal antibody. *Br. J. Cancer*, **57**, 239.

JHA, R.S., WICKENDEN, C., ANDERSON, M.C. & COLEMAN, D.V. (1984). Monoclonal antibodies for the histopathological diagnosis. *Br. J. Obstet. Gynaecol.*, **91**, 483.

JOHNSON, P.M., CHENG, H.M., MOLLOY, C.M., STERN, M.M. & SLADE, M.B. (1981). Human trophoblast specific surface antigen identified using monoclonal antibodies. *Am. J. Reprod. Immunol.*, **1**, 218.

JOHNSON, P.M. (1984). Immunobiology of human trophoblast. In *Immunological Aspects of Reproduction in Mammals*, Creighton, D.B. (eds). p. 109. Butterworths: London.

KAWANA, T. & YUSHINO, K. (1980). Estimation of type specific neutralizing antibody to HSV II. *Microbiol. Immunol.*, **24**, 1163.

LINDGREN, J., VESTERINEN, E., PUROLA, E. & WASHINGTON, T. (1985). Prognostic significance of tissue CEA in mild dysplasia of the uterine cervix. *Tumour Biol.*, **6**, 465.

McCANCE, D.H., CAMPION, M.J., CLARKSON, P.L. & CHESTERS, P.M. (1985). HPV 16 DNA sequences in CIN and invasive carcinoma of cervix. *Br. J. Obstet. Gynaecol.*, **92**, 1101.

McDICKEN, I.W., McLAUGHLIN, P.J., TROMANS, P.M., LUESLEY, D.M. & JOHNSON, P.M. (1985). Detection of placental-type alkaline phosphatase in ovarian cancer. *Br. J. Cancer*, **52**, 59.

McDICKEN, I.W., STAMP, G.H., McLAUGHLIN, P.J. & JOHNSON, P.M. (1983). Expression of human placental-type alkaline phosphatase in primary breast cancer. *Int. J. Cancer*, **32**, 205.

McLAUGHLIN, P.J., WARNE, P.H., HUTCHINSON, G.E., JOHNSON, P.M. & TUCKER, D.F. (1987). Placental-type alkaline phosphatase in cervical neoplasia. *Br. J. Cancer*, **55**, 197.

SIKORA, K., SMEDLEY, H. & THORPE, P. (1984). Tumour imaging and drug targeting. *Br. Med. Bull.*, **40**, 233.

SMITH, N.C., BRUSH, M.G. & LUCKETT, S. (1974). Preparation of human placental villous surface membrane. *Nature*, **252**, 302.

SOUTHALL, P.J., BOXER, G.M., BAGSHAWE, K.D., HOLE, N., BROMLEY, M. & STERN, P.L. (1990). Immunohistological distribution of 5T4 antigen in normal and malignant tissues. *Br. J. Cancer*, **61**, 89.

STERN, E. & NEELY, P.M. (1964). Dysplasia of the uterine cervix, incidence of regression, recurrence and cancer. *Cancer*, **17**, 508.

TRAVERS, P. & BODMER, W. (1983). Preparation and characterisation of monoclonal antibodies against PLAP and other human trophoblast associated determinants. *Int. J. Cancer*, **33**, 633.

VILLARD, L., MURPHY, M. & VESSEY, M.P. (1989). Cervical cancer deaths in young women. *Lancet*, **i**, 377.